# The Effects of Expressive Arts Therapy on Emotion Regulation and Aggression in Violent Offenders: A Randomized Controlled Trial

**DOI:** 10.3390/bs16081366

**Published:** 2026-08-10

**Authors:** Zhijie Xu, Xixi Ou, Jialin Fan

**Affiliations:** 1 School of Psychology, Shenzhen University, Shenzhen 518052, China; 2022341092@email.szu.edu.cn (Z.X.); 2020123037@email.szu.edu.cn (X.O.); 2 School of Artificial Intelligence, Shenzhen University, 518060, Shenzhen, China; 3 Shenzhen Humanities and Social Sciences Key Research Bases of the Center for Mental Health, Shenzhen 518060, China

**Keywords:** expressive arts therapy, violent offenders, emotion regulation, cognition reappraisal

## Abstract

Violent crime is a widely recognized public health concern. According to emotion regulation theory, aggressive behavior in violent offenders is linked to maladaptive emotion regulation strategies. However, few studies have explored interventions targeting emotional factors to reduce aggression in this population. Expressive arts therapy (EAT), which facilitates emotional expression and release, has shown therapeutic potential, but its effectiveness for violent offenders remains understudied. This randomized controlled trial examined the effects of an 8-week EAT program on emotion regulation and aggressive behavior in male violent offenders from a prison in China. Participants were assigned to either the intervention group (*n* = 31) or the control group (*n* = 34). Outcomes were assessed using cognitive reappraisal tasks, the Implicit Association Test, visual analogue mood scales, and the Emotion Regulation Strategies for Artistic Creative Activities Scale. Key findings revealed that, after 4 weeks of intervention, the experimental group demonstrated significantly improved cognitive reappraisal abilities and weakened “violence-positive” implicit associations, suggesting that enhanced cognitive reappraisal may be one key mechanism through which EAT operates. These results suggest that EAT effectively fosters positive psychological resources (i.e., cognitive reappraisal) and promotes non-violent implicit attitudes among violent offenders. This study provides initial quantitative evidence for EAT as a promising intervention for reducing aggression-related cognitive and implicit processes. Future research should further investigate its long-term effects and underlying mechanisms.

## 1. Introduction

Violence and criminal behavior are now widely recognized as significant global public health concerns (Glenn & Raine, 2014). Defined as the intentional use of physical force or power—whether threatened or actual—that results in or is likely to cause physical injury, death, or psychological harm (Quinsey et al., 2006), violent crime presents complex challenges across societies. Empirical evidence demonstrates that violent recidivism can be predicted with substantial accuracy among male offenders with prior violent histories (Rice, 1997). Understanding the underlying causes of violent behavior is essential for developing effective prevention and intervention strategies.

Emotion has been recognized as a key factor in aggression for a long time. According to the revised frustration-aggression theory, frustration indirectly influences aggression through negative emotions, with the intensity of aggression tendencies moderated by higher-order cognitive processes like attributions and social norms (Berkowitz, 1989). Meanwhile, traditional self-control theory suggests that negative emotions lead to aggressive behavior through impairing self-regulation (Leith & Baumeister, 1996). While these theories differ in focus, both highlight the role of negative emotions in aggression.

Emotion regulation theory (Gross, 1999) provides a parsimonious and unifying framework for understanding the aggression-emotion nexus. According to the process model, negative emotions create a motivation to regulate, and aggression can serve as a maladaptive coping strategy when adaptive strategies—particularly cognitive reappraisal—are deficient or unavailable (Chester & DeWall, 2017). Cognitive reappraisal, defined as the capacity to reinterpret the meaning of emotional stimuli to alter their emotional impact, is identified as a cornerstone of healthy emotional functioning (Gross & John, 2003). Deficits in this specific capacity have been consistently documented among violent offenders (Gillespie et al., 2018; Tonnaer et al., 2017), making it a precise and theory-grounded target for intervention.

Before proceeding, it is important to clarify that in this manuscript, affect refers to basic, neurobiologically primitive feeling states characterized by valence and arousal (Russell, 2003), whereas emotions are more complex, object-directed responses involving cognitive appraisal of specific events (Gross, 2015; Scherer, 2005). We use the term ‘emotion regulation’ to refer to the higher-order cognitive processes assessed in this study, while acknowledging that affective processes may play a significant underlying role.

Previous research has found emotional problems in violent offenders. These individuals tend to use poor emotion regulation strategies and have trouble recognizing their own emotions (Roberton et al., 2012, 2014). Compared with non-violent criminals or non-crime individuals, violent offenders have different facial emotions perception (Högman et al., 2020; Zeng et al., 2022), especially showing a bias toward interpreting expressions as angry, which increases feelings of hostility (Mellentin et al., 2014). When provoked, violent offenders typically struggle more with emotional control (Tonnaer et al., 2017). According to emotion regulation theory, aggressive behavior reflects a maladaptive attempt to modulate negative affective states (Gross, 1999). However, within the highly structured and emotionally constrictive environment of a prison—where autonomy is limited, emotional expression is formally and informally suppressed, and confrontational interactions are prevalent—aggression may also represent a context-dependent, albeit destructive, coping strategy for regaining a sense of control or discharging tension (Gillespie et al., 2018). This environmental contingency underscores the importance of interventions that do not merely suppress aggression, but provide alternative, institutionally acceptable channels for emotional processing.

Notably, the development of cognitive reappraisal capacity may represent a crucial target for intervention, as this adaptive strategy enables individuals to transform emotional experiences through cognitive reframing rather than merely suppressing emotional responses. The cultivation of this positive psychological resource may be particularly valuable for populations characterized by emotional dysregulation.

However, few studies have explored interventions targeting emotional issues in violent offenders, even though this appears to be a promising approach for reducing relapse. Existing interventions for violent offenders mainly focus on cognitive-behavioral therapy (CBT). However, because of complex needs in incarcerated individuals, this individualized approach places high demands on therapists (Papalia et al., 2019). Other intervention methods have shown mixed results. One randomized controlled trial tested emotion recognition training for adult violent offenders. While it effectively reduced their tendency to perceive anger in ambiguous facial expressions, it did not meaningfully reduce aggression (Kuin et al., 2020). A virtual reality (VR)-based intervention improved emotion recognition but did not assess changes in aggressive behavior (Seinfeld et al., 2021). Another small-scale VR study reported improvements in aggression, emotion dysregulation, and anger expression, but lacked a control group (Ivarsson et al., 2023). Additionally, VR-based interventions face challenges in widespread implementation due to their high cost. These limitations call for alternative approaches that are group-deliverable, accessible to individuals with low verbal ability, and non-confrontational in nature. Expressive arts therapy (EAT) may address this need.

Expressive arts therapy (EAT) is a multimodal psychological approach that integrates various art forms—such as painting, music, dance, drama, and expressive writing—to facilitate psychological healing, resolve inner conflicts, and enhance self-awareness (Malchiodi, 2003). Grounded in the principle that creative expression can bypass verbal defenses and promote mental well-being, EAT operates through mechanisms like catharsis and the development of non-verbal expression channels (Lavania & Ballal, 2025). Critically, we propose that EAT specifically enhances cognitive reappraisal by allowing offenders to externalize emotional experiences into art and then re-examine their creations from different perspectives, thereby reconstructing the meaning of emotional events and developing less hostile interpretations (Levine et al., 2004). This reappraisal process is a core resource for healthy emotional functioning. Furthermore, drawing on the Extended Process Model (Gross, 2015), which emphasizes flexible strategy deployment across contexts, EAT incorporates varied artistic media and reflective exercises to foster regulatory flexibility—an ability particularly valuable in the changing demands of prison life. As a non-confrontational, group-based modality, EAT offers unique advantages for fostering emotion regulation, which established positive effects across diverse populations (Ho et al., 2020; Yan et al., 2021).

In Chinese correctional settings, EAT’s non-verbal, indirect modality may be particularly fitting: East Asian collectivist norms favor suppression over disclosure (Hu et al., 2014; Kraus et al., 2024; Tamir et al., 2024), a pattern linked to the high alexithymia rates in our sample and to interdependent self-construal. While alexithymia is primarily conceptualized as a stable personality trait (Taylor et al., 1997), its measured severity can be significantly influenced by contextual factors. The emotionally constrictive prison environment, which systematically penalizes emotional expression, may exacerbate the manifestation of alexithymic traits, potentially contributing to the elevated scores observed in correctional populations. Furthermore, East Asian cultural norms that favor emotional restraint may interact with this environmental pressure, amplifying the observed levels of alexithymia. Rather than posing a barrier, this cultural backdrop renders EAT a potentially congruent pathway that bypasses verbal prohibitions while enabling cognitive reappraisal through symbolic distancing and creative re-framing (Levine et al., 2004). Additionally, EAT’s emphasis on experiential over verbal processes enhances accessibility for individuals with limited education or resistance to talk therapy—advantages particularly salient in culturally suppressive and resource-constrained custodial contexts.

Despite these theoretical merits, empirical research on EAT in correctional populations remains scarce: no randomized controlled trial has been conducted, the specific mechanisms have not been empirically tested, and its cultural fit in non-Western prison settings has been assumed rather than systematically examined.

To address these gaps, this study aimed to examine the effects of EAT on emotion regulation among violent offenders through a controlled experiment. The participants were incarcerated violent offenders from a prison. The experimental group underwent an 8-week EAT group intervention, while the control group received only routine prison management. Beyond testing efficacy, we aimed to understand mechanisms of change within a culturally suppressive, resource-limited setting. Specifically, we predicted that (a) the EAT group would show significantly greater improvement in cognitive reappraisal task performance compared to the control group; (b) this improvement would be accompanied by a reduced “violence-positive” implicit attitude. Emotional states (VAMS) and art-specific regulation strategies (ERS-ACA) were examined as secondary, exploratory outcomes.

## 2. Materials and Methods

### 2.1. Participants

In January 2024, we obtained initial ethics approval for the observational questionnaire phase of this study. Following the required institutional review by the prison authorities, we distributed 440 paper questionnaires to violent offenders convicted of crimes including intentional homicide, intentional injury, affray, and robbery at a male prison in mid-January 2024. A total of 408 questionnaires were returned, and after excluding invalid responses with incomplete answers or excessively identical response patterns, 365 valid questionnaires were obtained. After analyzing the questionnaire data, we decided to conduct an intervention study and submitted a separate application for ethics approval specifically for the intervention component. This supplementary approval was granted in September 2024. From this pool, participants aged 20–58 years old with remaining sentences exceeding 6 months and demonstrating high alexithymia and difficulties in emotion regulation tendencies were selected, while excluding those with antisocial personality disorder or current mental illness diagnoses. Then, based on a willingness survey, 13 participants were excluded due to their unwillingness. Ultimately, the remaining participants were randomly assigned to either the intervention group (*n* = 31; mean age = 35.84 ± 7.89) or the control group (*n* = 34; mean age = 39.68 ± 10.86). Based on a power analysis using G*Power 3.1 software, with an effect size of f = 0.25, α = 0.05, and power = 0.80, the required sample size for a repeated-measures ANOVA was 28 participants. A total of 65 participants were ultimately included in this study, meeting the statistical requirements.

All participants were male and provided written informed consent after receiving a thorough explanation of the study purpose, procedures, potential risks and benefits, and their rights. Participants were explicitly informed that their participation was entirely voluntary, that they could withdraw at any time without penalty or negative consequences for their prison status or sentence, and that refusal to participate would not affect their access to routine prison services. Opt-out procedures were available at every session; participants who wished to discontinue were permitted to leave the group and return to their regular prison activities without any adverse consequences.

### 2.2. Measures

The primary outcome of this study was cognitive reappraisal capacity, as assessed by the picture-rating task. The secondary outcomes included implicit violent attitudes (IATs), mood states (VAMSs), and emotion regulation strategies during art creation (ERS-ACA). The selection of cognitive reappraisal as the primary outcome was theory-driven: our hypothesis centered on the mechanism of cognitive change through EAT. Alexithymia was selected as a screening criterion because (a) it is highly prevalent among violent offenders (approximately 82% in our sample); (b) it represents a core difficulty in emotion identification and verbal expression that is theoretically relevant to both emotion regulation deficits and aggression; and (c) EAT’s non-verbal approach is particularly well suited to addressing alexithymic traits, which are less amenable to verbal therapies. Thus, the selection of individuals with elevated alexithymia was intentional and theory-consistent.

#### 2.2.1. Screening Questionnaires

We measured aggression tendency, alexithymia, and difficulties in emotion regulation in the screening questionnaires.

The Buss & Perry Aggression Questionnaire (BPAQ-SF), developed by Buss and Perry (Diamond & Magaletta, 2006), was adopted to measure aggression tendency. The Chinese version of the questionnaire was revised by Luo Guiming (Luo, 2008). This scale consists of four dimensions with a total of 29 items. It is scored on a five-level scale ranging from 1 (completely inconsistent) to 5 (completely consistent). The statistical indicator is the total score. The higher the score, the stronger the aggressiveness. The scale demonstrated excellent internal consistency in this study (Cronbach’s α = 0.920).

To measure alexithymia, we adopted the 20-item Toronto Alexithymia Scale (TAS-20), developed by Taylor and revised by Bagby et al. The Chinese version was localized by Yi Jinyao et al. (Jinyao et al., 2003). This 20-item instrument assesses alexithymia across three dimensions: difficulty identifying feelings, difficulty describing feelings, and externally oriented thinking. All items are rated on a 5-point Likert scale ranging from 1 (strongly disagree) to 5 (strongly agree), with 15 positively worded items and 5 reverse-scored items. The total score serves as the primary metric, where higher scores indicate greater alexithymia severity. Based on established cutoffs, scores of 0–51 indicate no alexithymia, 52–60 suggest alexithymia tendencies, and scores ≥61 signify clinically significant alexithymia. In the current study, the scale demonstrated good internal consistency with a Cronbach’s α coefficient of 0.800.

We used the Brief Version of the Difficulties in Emotion Regulation Scale (DERS-16) to measure emotion regulation difficulties, which consists of 16 items across five dimensions (Bjureberg et al., 2016). Items are rated from 1 (“almost never”) to 5 (“almost always”). The total score indicates emotion regulation difficulties, with higher scores reflecting greater difficulties. In this study, the scale showed excellent reliability (Cronbach’s α = 0.954).

#### 2.2.2. Cognitive Reappraisal

Cognitive reappraisal was assessed via a picture-rating task utilizing 40 negative images selected from the Chinese Affective Picture System (Bai et al., 2005). The task consisted of two parts: “rating after viewing” and “rating after cognitive reappraisal”, with 20 randomly assigned images for each part. Participants first completed the “rating after viewing” section, followed by the “rating after cognitive reappraisal” section, where they were instructed to provide positive interpretations of the depicted persons or scenarios before rating their emotional response on a scale from 1 (minimal negative emotion) to 9 (strongest negative emotion). The task was programmed using E-Prime 3.0. For the second and third administrations of the cognitive reappraisal assessment, half of the images were replaced compared to the previous session, while both control and experimental groups viewed the same set of images each time.

While this task provides a controlled measure of instructed reappraisal capacity, we acknowledge that it may not fully capture spontaneous use of reappraisal in real-world contexts, nor does it assess the broader construct of emotion regulation flexibility. The task is best understood as an indicator of capacity for cognitive reappraisal under structured conditions, rather than as a comprehensive measure of emotion regulation ability.

#### 2.2.3. Implicit Violent Attitudes

Greenwald’s Implicit Association Test (IAT) was applied to measure implicit violent attitudes, using two types of implicit word associations: “self-violence” and “violence-positive” (Xiang et al., 2014). The “self-violence” IAT assessed individuals’ automatic associations between self-concept and violence by measuring reaction times when pairing self-related words (e.g., “I”, “myself”, “he”, “others”) with violence-related attributes (e.g., “war”, “fight”, “peace”, “friendly”). The “violence-positive” IAT evaluated implicit attitudes toward violence by measuring association speeds between violence-related words (e.g., “hit”, “attack”, “defend”, “dodge”) and valenced attributes (e.g., “reward”, “wise”, “punish”, “failure”). The test included four critical blocks: block 4 (violence-self + peace-others pairing), block 7 (violence-others + peace-self pairing), block 11 (positive-defend + negative-attack pairing), and block 14 (positive-attack + negative-defend pairing). The IAT task was programmed using E-Prime 3.0.

#### 2.2.4. Mood States

We employed the Visual Analogue Mood Scale (VAMS) to assess participants’ mood states, which consists of 18 items across four dimensions. Each item presents a pair of antonymous adjectives, and participants rate their current state by clicking on a 100-point continuum between the two descriptors (e.g., for the first item pair, selecting 1 indicates “extremely drowsy” while 100 represents “extremely alert”). The scale includes 13 forward-scored and 5 reverse-scored items, measuring four dimensions: somatic state, psychosomatic state, emotional state, and extraversion. Higher composite scores indicate greater positivity in each respective dimension. The assessment was programmed using NET 8.0.

#### 2.2.5. Emotion Regulation Strategy

This study utilized the Emotion Regulation Strategies for Artistic Creative Activities Scale (ERS-ACA) to assess participants’ emotion regulation strategies during art creation. The Chinese version was developed through a rigorous translation process involving English majors, psychology students, and faculty members who conducted forward and backward translations before finalizing the Chinese adaptation. The 18-item scale employs a 5-point Likert scale (1 = strongly disagree to 5 = strongly agree) across three dimensions: Avoidance Strategies, Approach Strategies, and Self-Development Strategies. Dimension scores are calculated as mean values, with higher scores indicating more positive regulation strategies. The scale demonstrated excellent internal consistency in this study (Cronbach’s α = 0.914).

#### 2.2.6. Expressive Art Therapy

The art therapy program was conducted by professionals specializing in psychodynamic and EAT research who held relevant qualifications in expressive arts therapy and had received training in delivering arts-based interventions in correctional settings. The study protocol was approved by the Institutional Review Board of Shenzhen University (SZU_PSY_2024_127) prior to participant recruitment. The therapy program consisted of eight sessions: Body and Emotions (using drawing to help participants perceive their bodies and recent emotions), Stress Volcano (using clay and painting tools to guide participants in coping with stress appropriately), Seeing Emotions (using painting to enhance participants’ intuitive recognition of emotions, others’ reactions to these emotions, and their own emotional experiences), Unspoken Words (using clay work to promote problem-solving and the transformation/sublimation of defenses), Self-Awareness (using painting to improve problem-solving and reappraisal abilities), Life Line (using clay work and painting to help participants re-examine past mistakes and reconstruct themselves with new perspectives and attitudes), Flow of Life (using painting and music to enhance participants’ awareness of their group roles, improve interpersonal skills, and strengthen emotional expression), and Review & Prospect. These processes, while not quantitatively analyzed in the current study, were theoretically intended to facilitate narrative revision and identity re-evaluation to encompass a broader process of meaning-making. For example, the ‘Life Line’ session required participants to use clay and painting to map significant life events—both positive and negative—along a temporal axis. They were then guided to reflect on the emotions attached to these events and to symbolically ‘re-shape’ or ‘re-interpret’ key turning points. The program lasted eight weeks, with one theme covered per week in two-hour sessions.

### 2.3. Procedures

A total of 31 participants in the intervention group were divided into three groups in this project, with approximately 10 participants per group.

**Figure 1:**
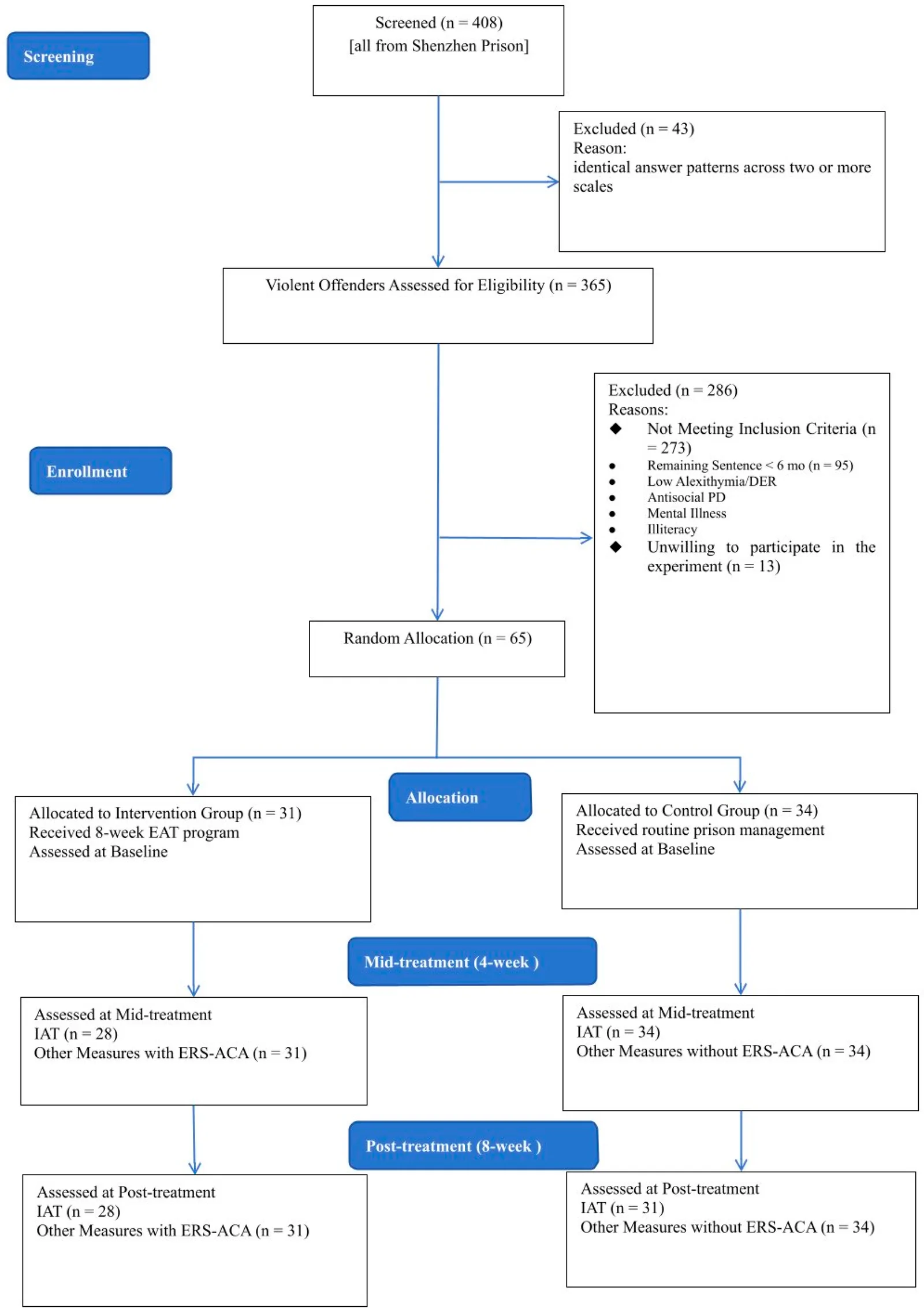
Study flow diagram. Note. This diagram illustrates the flow of participants from initial screening through the 8-week intervention period. A total of 408 violent offenders from one prison were screened. Forty-three were excluded due to invalid questionnaires (identical answer patterns). Of the remaining 365 assessed for eligibility, 286 were excluded for not meeting inclusion criteria (e.g., short remaining sentence, low alexithymia/emotion regulation difficulties, antisocial personality disorder, mental illness, illiteracy) or being unwilling to participate. Sixty-five eligible participants were randomly allocated to either the expressive arts therapy (EAT) intervention group (*n* = 31) or the treatment-as-usual control group (*n* = 34). Both groups were assessed at baseline. The intervention group completed the 8-week EAT program, with assessments conducted at mid-treatment (4 weeks) and post-treatment (8 weeks) using the Implicit Association Test (IAT, *n* = 28) and other measures, including the Emotion Regulation Strategies for Artistic Creative Activities scale (ERS-ACA, *n* = 31). The control group received routine prison management.

All participants completed the picture-rating task, IAT, and VAMS at three time points: baseline (before the intervention), after four weeks of sessions, and upon completion of the entire intervention program. Specially, ERS-ACA scales were administered after four weeks of sessions and at the end of the intervention sessions only in the intervention group. The participant flow throughout this study is illustrated in Figure 1.

While the control group received routine prison management without the EAT intervention, this design was approved by the IRB on the grounds that (a) EAT is an adjunctive, non-essential therapeutic activity rather than a standard clinical treatment for this population; (b) the prison’s routine psychological services remained available to all participants; and (c) this study employed a conservative approach to introducing a novel intervention, which is appropriate given the limited prior evidence for EAT in this setting. Nevertheless, we acknowledge that a waitlist control design would have been ethically preferable.

### 2.4. Statistical Analysis

SPSS 26.0 was used to carry out all the statistical analyses. Descriptive statistics are reported as mean ± standard deviation (SD). Pearson correlation analyses of baseline aggression tendency, alexithymia, and emotion regulation difficulties were conducted.

The IAT data were processed according to the following criteria: (a) exclusion of participants with missing data in any of the three experimental sessions; (b) removal of participants with more than 10% of trials showing RTs > 10,000 ms or <300 ms; (c) exclusion of the first two trials from each block; (d) elimination of data with mean error rates exceeding 20%; and (e) replacement of error trial RTs with the corresponding block mean plus 600 ms. After data cleaning, the final sample sizes were 28 and 31 for the experimental and control groups, respectively. We then calculated the “violence-self” IAT effect by subtracting the mean RT of Block 4 from that of Block 7 and similarly calculated the “violence-positive” IAT effect by subtracting the mean RT of Block 11 from that of Block 14. To examine the effects of art therapy courses on implicit violent attitudes in violent offenders, separate 2 × 3 repeated-measures ANOVAs were conducted for the violence-self IAT and the violence-positive IAT.

To assess the impact of EAT on cognitive reappraisal in violent offenders, a 2 × 2 repeated-measures ANOVA was conducted. Similarly, repeated-measures ANOVAs were also performed for the VAMS and ERS-ACA data. All statistical tests were two-sided, with a *p*-value < 0.05 considered statistically significant.

## 3. Results

### 3.1. Descriptive Statistics and Correlation Results at Baseline

The means and standard deviations of the demographic variables and screening questionnaires are reported in Table 1. The scores of BPAQ-SF, TAS-20, and DERS-16 were all higher than those in the normative population. A total of 82% of participants met the diagnostic criteria for alexithymia. The results revealed significant positive correlations between aggression tendency, emotion regulation difficulties, and alexithymia among violent offenders, as detailed in Table 2.

Among different time points, the descriptive statistics of cognitive reappraisal and IAT are presented in Table 3. The results of VAMS and ERS-ACA are reported in Table 4 and Table 5, respectively.

### 3.2. Cognitive Reappraisal

The results revealed a significant main effect of group (F = 4.133, η^2^ = 0.004, *p* < 0.05), as well as a significant group × time interaction effect (F = 18.129, η^2^ = 0.017, *p* < 0.05). At the 4-week assessment, the experimental group (M = 4.44, SD = 2.70) showed significantly better outcomes than the control group (M = 5.38, SD = 3.14). However, by the 8-week follow-up, no significant between-group differences in scores were observed.

### 3.3. Implicit Violent Attitudes

For the violence-self IAT, the main effect of time was not significant, while the main effect of group was significant (F(1, 1042) = 5.993, η^2^ = 0.005, *p* < 0.05) and the time × group interaction was significant (F(2, 2084) = 3.680, η^2^ = 0.004, *p* < 0.01). Simple effects analysis revealed a significant difference between the experimental and control groups at 4 weeks (F(1, 1042) = 4.358, η^2^ = 0.004, *p* < 0.05), but no significant between-group difference was found at 8 weeks. Similarly, for the violence-positive IAT, neither the main effect of time nor group was significant, but the time × group interaction reached significance (F(2, 2084) = 4.864, η^2^ = 0.005, *p* < 0.01), with follow-up analyses showing a significant group difference at 4 weeks (F(1, 1042) = 7.754, η^2^ = 0.007, *p* < 0.01) that disappeared by 8 weeks.

## 4. Discussion

This RCT provides the first controlled evidence that EAT’s effect on aggression-related cognition in violent offenders operates specifically through enhanced cognitive reappraisal. At the 4-week assessment, the EAT group demonstrated significantly improved cognitive reappraisal capacity and weakened “violence-positive” implicit associations compared to controls. This pattern suggests that the cultivation of cognitive reappraisal—the capacity to reframe emotional triggers (Gross, 1999)—serves as a key mechanism through which EAT may reduce the appeal of aggression as a regulatory alternative (Webb et al., 2012). In doing so, our findings extend prior research that focused on emotion recognition training with mixed results (Kuin et al., 2020), indicating that emotion regulation capacity, rather than emotion perception, may be the more critical intervention target for this population.

This pattern of results is coherent with the process model’s theoretical prediction (Gross, 1999): when individuals acquire a more adaptive regulatory strategy, the perceived utility of aggression as a regulatory alternative diminishes. Cognitive reappraisal operates by altering the meaning of emotional stimuli before a full affective response is generated, thereby reducing the motivational pressure to discharge negative affect through impulsive actions (Gross & John, 2003). In the context of violent offenders, many of whom have relied on aggression as a primary means of coping with provocation (Roberton et al., 2012), the acquisition of reappraisal skills may disrupt this ingrained response pattern by offering a non-destructive alternative. Meta-analytic evidence confirms that cognitive reappraisal is among the most effective strategies for attenuating negative emotional responses (Webb et al., 2012), and our findings suggest that EAT provides a viable pathway for cultivating this capacity in a population where verbal therapies often face engagement barriers. Notably, this mechanism-based interpretation advances the literature beyond prior studies that reported EAT’s effects without specifying the psychological processes through which they emerged.

Regarding the Implicit Association Test results, the weakened “violence-positive” association demonstrates art therapy’s significant impact on reducing offenders’ aggressive tendencies. This test can reveal outcomes that might not emerge in direct surveys (Arnoult et al., 2023), making its results particularly reliable. After art therapy intervention, offenders showed reduced automatic associations between violence and positive concepts, suggesting decreased preference for violent solutions. Previous research suggests that violence is often associated with positive affect because it can serve as a short-term problem-solving strategy, despite its long-term negative consequences (Chester, 2017; Chester & DeWall, 2017). The weakened violence-positive association implies offenders may be less likely to resort to violence in the future. The development of cognitive reappraisal as an internal resource likely contributes to this shift, as individuals who can effectively reframe a situation may find less appeal in violent solutions. These findings complement the cognitive reappraisal results, collectively highlighting emotion regulation as a promising intervention approach for violent offenders.

However, an unexpected outcome was observed in the IAT results at 4-week: EAT actually strengthened the “violence-self” association among violent offenders. From the perspective of psychodynamic extensions of the emotion regulation framework, this may reflect a temporary “defensive disruption” phase (Shedler, 2010). In this view, the creative and symbolic nature of EAT might have lowered psychological resistance, allowing participants to more honestly confront and externalize their self-concept as “violent”—a necessary, albeit uncomfortable, precursor to reappraisal. However, we emphasize that this interpretation is post-hoc and theory-generating rather than confirmatory. Alternative explanations, such as task-specific priming effects or demand characteristics, cannot be ruled out and warrant systematic investigation in future studies.

The attenuation of all significant effects at the 8-week assessment warrants careful consideration. Several non-mutually-exclusive explanations exist. First, the observed pattern may reflect the genuine transience of EAT effects: without continued practice and reinforcement, improvements in cognitive reappraisal may not be sustained. Second, the duration of the intervention (8 weeks) may be insufficient to alter deeply entrenched emotional patterns. Critically, alexithymia was not reassessed post-intervention, preventing us from testing whether EAT influenced alexithymia itself. This design gap leaves unclear whether EAT produced genuine trait-level change or merely altered state-dependent symptom expression—a distinction that future research must address with repeated alexithymia assessments. Third, methodological confounds cannot be ruled out: repeated testing may have induced fatigue, and the Spring Festival holiday (falling during the 8-week assessment period) may have introduced situational emotional vulnerability unique to incarcerated individuals. The present data do not permit adjudication between these competing accounts. We therefore view the 8-week null findings not as a definitive conclusion about EAT’s long-term ineffectiveness, but as a critical empirical boundary condition that future studies should systematically investigate through extended follow-up designs and the inclusion of maintenance interventions.

The intervention was conducted in a Chinese prison setting, where cultural influences on emotion regulation warrant consideration. Prior research indicates that East Asian collectivist cultures tend to favor emotion suppression over expressive disclosure (Hu et al., 2014; Ramzan & Amjad, 2017), a pattern linked to interdependent self-construal and psychological well-being in these contexts (Tamir et al., 2024; Kraus et al., 2024). Based on this, we reasoned that EAT’s non-verbal, indirect approach might offer a culturally congruent pathway for emotional exploration. However, a key limitation of the present study is that we did not directly measure participants’ interdependent self-construal, precluding empirical tests of this cultural-fit assumption or confirmation that EAT’s effects operated through culturally compatible mechanisms. Thus, while we observed positive effects of EAT, whether these effects were moderated by individual cultural orientation or mediated by interdependence remains unknown. Future research should incorporate cultural variables—particularly interdependent self-construal—to clarify the boundary conditions and mechanisms underlying EAT’s efficacy across diverse cultural groups.

No significant changes were observed in ERS-ACA or mood states (VAMS) at either week 4 or week 8. ERS-ACA scores remained consistently high, showing no statistically significant improvement beyond the initial measurement. Although minor increases were noted in some dimensions by week 8, these changes were not significant. The lack of significant change in ERS-ACA may be attributed not only to limited sample size and statistical power but also to potential ceiling effects, as it maintained high scores since the first measurement. This raises the question of whether the scale adequately captures changes in emotion regulation within artistic contexts for this specific population, or whether its sensitivity is limited when participants already exhibit high initial levels. Future studies could incorporate qualitative methods, such as post-session interviews, to triangulate and enrich findings regarding how participants regulate emotions during art-making.

Similarly, VAMS scores remained stable and relatively negative throughout this study, likely reflecting the highly structured and restrictive prison environment rather than treatment effects. As VAMS assesses transient mood states susceptible to situational influences, its utility for evaluating sustained intervention effects in correctional settings may be limited. Future research should include trait-based measures of emotional functioning—such as follow-up assessments using the DERS-16—to more accurately capture lasting treatment outcomes (Bjureberg et al., 2016).

Compared to CBT, EAT demonstrates comparable effectiveness while offering unique advantages for prison settings: better suitability for group formats, non-verbal expression options, and enhanced social support (Kim, 2010; Levine et al., 2004). Considering prisoners’ typically low education levels and high alexithymia rates, verbal therapies may have a limited impact. Arts therapy’s non-verbal approach proves particularly valuable here. It may provide an alternative pathway to access and develop cognitive-emotional resources like reappraisal, which might be less accessible through purely verbal means. Long-term benefits may include improved self-awareness, creative self-expression, and empowerment. These advantages make EAT particularly promising for this population.

However, we should acknowledge that any intervention within a total institution like a prison inevitably operates at the intersection of care and control. While we demonstrate positive psychological effects, it is important to consider that EAT programs, despite their rehabilitative intent, may also serve institutional aims by enhancing behavioral compliance and reducing disruptions. This dual-use potential raises ethical questions about the conditions under which these programs are offered and whether they are genuinely participant-centered or primarily serve the institution’s interests. The present study is not designed to answer these questions, but future research and practice should remain reflexively aware of the power dynamics inherent in correctional rehabilitation.

Several limitations should be noted. Most critically, while our findings suggest that EAT may reduce aggression-related cognitive and implicit processes, we did not measure actual aggressive behavior. Our outcome measures—cognitive reappraisal task performance and IAT scores—are proxies for emotion regulation capacity and implicit attitudes, respectively, rather than direct indicators of violence or aggression. Statements derived from these measures must therefore be interpreted with caution, and future research should incorporate longitudinal behavioral indicators such as institutional misconduct records and post-release recidivism data. Second, the generalizability of our findings is constrained by the highly selective nature of the sample. Participants were not representative of all violent offenders; rather, they represent a subset characterized by elevated alexithymia and emotion regulation difficulties who were willing to participate in an arts-based intervention. The findings may not extend to violent offenders without these characteristics, to those who decline such interventions, or to offenders in non-Chinese correctional systems. Third, we lacked long-term follow-up data. Fourth, relying solely on self-report measures administered intensively may have caused fatigue. Future research could streamline assessments and incorporate physiological measures.

Furthermore, a significant limitation of the present study is the absence of data on the artistic process and the participants’ lived experiences. Despite delivering an arts-based intervention, our analysis relied exclusively on quantitative outcomes. Consequently, we cannot report on the thematic content of the artwork, participants’ interpretations of their creations, or their subjective accounts of personal change. This methodological choice, while appropriate for establishing initial efficacy, leaves the ‘black box’ of EAT largely unopened. Furthermore, while we frame the improvement in cognitive reappraisal as a key mechanism, it is likely that EAT’s benefits extend beyond this single pathway. The intervention included components explicitly designed for narrative reflection (e.g., the ‘Life Line’ session), which resonate with broader theoretical frameworks such as desistance theory and narrative criminology (Cheliotis & Jordanoska, 2016). These perspectives posit that successful rehabilitation involves ‘secondary desistance’—a fundamental shift in self-identity and self-narrative (Maruna & Farrall, 2004). The symbolic re-working of personal histories through art contributed to a more prosocial identity, increased agency, and a more hopeful future outlook. Consequently, the present study is well-positioned to demonstrate short-term cognitive and implicit changes, but it remains agnostic about whether, or how, these changes translate into the identity-level transformations. Future research must prioritize mixed-methods designs, incorporating art-based assessments and narrative interviews to capture the identity-related, agentic, and existential dimensions of change.

Future research should incorporate longitudinal behavioral indicators such as institutional misconduct records, incident reports of physical altercations, and—critically—post-release recidivism data to substantiate claims about actual violence reduction. Without such evidence, the present findings should be viewed as demonstrating changes in cognitive and implicit processes that are theoretically linked to, but not synonymous with, aggression. Future research should also examine long-term outcomes and mechanisms, potentially incorporating electrophysiological measures. Moreover, combining arts therapy with other evidence-based approaches (Papalia et al., 2019) may enhance effectiveness. Considering scalability, developing simplified, staff-led protocols could facilitate broader implementation. In addition, future research could incorporate physiological measures (e.g., heart rate variability) to examine whether EAT’s effects involve autonomic nervous system changes, as suggested by polyvagal theory (Porges, 2011).

## 5. Conclusions

This study suggests that EAT may effectively reduce aggression in violent offenders in the short term (4 weeks), primarily by enhancing cognitive reappraisal, and may promote non-violent implicit attitudes, thereby supporting our main hypothesis. However, the long-term effects (8 weeks) were not significant. This study provides initial evidence for EAT as a promising intervention for reducing aggression-related cognitive and implicit processes. Future research should further investigate its long-term effects and underlying mechanisms, incorporating longitudinal behavioral outcomes.

## Figures and Tables

**Table 1:** Descriptive statistics for demographic variables and screening questionnaires.

	Control Group (*n* = 34) (M ± SD)	Intervention Group (*n* = 31) (M ± SD)
Age	39.68 ± 10.86	35.84 ± 7.89
Remaining sentence length	1.91 ± 0.29	1.71 ± 0.461
Education level ^a^	1.91 ± 0.83	2.29 ± 0.86
BPAQ-SF	80.74 ± 16.65	85.55 ± 12.84
TAS-20	56.74 ± 7.02	56.26 ± 7.55
DERS-16	28.94 ± 10.15	30.81 ± 8.99

Note. BPAQ-SF, the Buss & Perry Aggression Questionnaire. TAS-20, the 20-item Toronto Alexithymia Scale. The same below. DERS-16, the Brief Version of the Difficulties in Emotion Regulation Scale. ^a^ Education level was scored on a 4-point scale: 1 = primary school or below, 2 = junior secondary school, 3 = high school/secondary technical degree, 4 = tertiary education or above.

**Table 2:** The correlation between emotion regulation difficulties, aggression tendency, and alexithymia.

	BPAQ-SF	TAS-20	DERS-16
BPAQ-SF	1		
TAS-20	0.580 **	1	
DERS-16	0.636 **	0.621 **	1

Note. **, *p* < 0.01.

**Table 3:** The descriptive statistics of cognitive reappraisal and IAT.

	Time	Control Group (M ± SD)	Intervention Group (M ± SD)
Cognitive reappraisal	4 weeks	5.38 ± 3.14	4.44 ± 2.70
	8 weeks	4.85 ± 3.06	4.60 ± 2.95
Self-violence IAT	0 weeks	−24.62 ± 357.61	38.94 ± 370.32
	4 weeks	8.26 ± 491.93	68.69 ± 443.26
	8 weeks	22.73 ± 508.709	6.92 ± 559.19
Violence-positive IAT	0 weeks	−160.85 ± 389.18	−153.55 ± 388.31
	4 weeks	−247.75 ± 704.69	−134.70 ± 606.02
	8 weeks	−145.10 ± 647.52	−179.09 ± 574.48

Note. IAT, Implicit Association Test. Data are presented as mean ± standard deviation. Cognitive reappraisal is measured in arbitrary units on a scale from 1 (minimal negative emotion) to 9 (strongest negative emotion) and the IAT effects are measured by the RT obtained after data processing. Measurements were taken at three time points: baseline (0 weeks), after the 4-week EAT program (4 weeks), and after the 8-week EAT program (8 weeks).

**Table 4:** Descriptive statistics for VAMS.

Time	Group	Physical State	Mental State	Emotion State	Extroversion
0 weeks	control	377.61 ± 135.347	129.33 ± 62.781	278.03 ± 106.998	36.12 ± 29.148
	intervention	357.10 ± 109.839	140.35 ± 59.862	301.42 ± 101.082	42.29 ± 32.412
4 weeks	control	341.52 ± 106.367	119.15 ± 68.392	299.52 ± 110.854	37.82 ± 28.390
	intervention	357.26 ± 81.750	148.55 ± 47.636	271.71 ± 67.936	44.94 ± 25.551
8 weeks	control	325.68 ± 119.005	130.07 ± 73.853	283.75 ± 108.640	46.79 ± 30.573
	intervention	347.77 ± 97.719	149.20 ± 64.245	265.87 ± 87.179	43.07 ± 30.112

Note. VAMS = Visual Analogue Mood Scales. Data are presented as mean ± standard deviation. All subscales (Physical State, Mental State, Emotion State, Extroversion) are measured in arbitrary units on a continuous scale, with higher scores indicating a more positive state or greater extroversion. Measurements were taken at three time points: baseline (0 weeks), after the 4-week EAT program (4 weeks), and after the 8-week EAT program (8 weeks).

**Table 5:** Descriptive statistics for ERS-ACA.

	Avoidance Strategies	Approach Strategies	Self-Development Strategies
4 weeks	3.197 ± 0.541	3.617 ± 0.559	3.473 ± 0.595
8 weeks	3.333 ± 0.372	3.789 ± 0.529	3.747 ± 0.6124

Note. Data are presented as mean ± standard deviation. Only the EAT group completed this scale. All subscales (Avoidance Strategies, Approach Strategies, Self-Development Strategies) are measured in arbitrary units on a 5-point Likert scale (1 = strongly disagree to 5 = strongly agree), with higher scores indicating a more positive state. Measurements were taken at two time points: after the 4-week EAT program (4 weeks), and after the 8-week EAT program (8 weeks).

## Data Availability

The data presented in this study are not publicly available due to ethical and privacy restrictions. This study was conducted with a vulnerable population—incarcerated violent offenders—within a correctional facility. Participants provided informed consent with the assurance that their individual data would remain confidential and would not be shared outside the research team. Furthermore, the prison administration imposed strict data governance requirements, including that all data remain under institutional control and be used solely for the purposes of this approved study. Access to the data is restricted to the principal investigators and authorized research personnel. De-identified data may be made available to qualified researchers upon reasonable request to the corresponding author (fanjl@szu.edu.cn), subject to approval from the Ethics Committee of Shenzhen University and the prison administration, and in compliance with applicable privacy and institutional regulations.
